# Selenium in sepsis – substitution, supplementation or pro-oxidative bolus?

**DOI:** 10.1186/cc13963

**Published:** 2014-07-02

**Authors:** Lutz Schomburg

**Affiliations:** 1 Institute for Experimental Endocrinology, Charité Medical School Berlin, CVK, Suedring 10, D-13353 Berlin, Germany

## 

Sakr and colleagues report seemingly disappointing data on the effects of selenium in severe sepsis [1]. Mortality rates remained unaffected despite intensive selenium supplementation. The authors conclude that extra selenium above a basal supply of 100 μg/day provides no additional health benefit.

However, there are good reasons for selenium supplementation in sepsis; the selenium status declines with the disease especially in nonsurvivors [2], reducing selenoprotein P levels [3], while supplemental selenium efficiently restores selenoprotein expression in experimental models [4].

Yet some major questions prevail. Is it sufficient to balance selenium decline (substitution)? Are larger dosages required for overcoming compromised selenoprotein biosynthesis (supplementation)? Are bolus dosages of selenite key to health benefits (pro-oxidative)? For me, the third option has been specifically disproven by the data [1].

So why does this study seemingly contradict the positive findings of Angstwurm and colleagues [5]? An answer may be deduced from the comparison of the selenium and selenoprotein concentrations before and during supplementation. However, these data are unfortunately not provided. The perception of selenium in medicine currently assumes a J-shaped curve relationship between intake and effects; that is, conferring health benefits upon supplementation only for the deficient individuals. We thus need to monitor the selenium status of critically ill patients to better understand its importance and to identify those patients in need of supplemental selenium. While the optimal dosage remains to be determined, a pro-oxidative bolus has been disqualified without calling selenium supplementation in dosages beyond pure substitution into question.

## Authors’ response

Yasser Sakr

We read with interest the letter by Dr Schomburg concerning our article published recently in *Critical Care*[1]. He interpreted the results of this study as disproving a possible beneficial effect of bolus selenite supplementation in patients with severe sepsis. However, the limitations of our study must be taken into consideration before reaching this conclusion. The retrospective design may have introduced bias by indication, with selenium given only to the more severely ill patients. In addition, some factors that may impact on outcomes were not included in the multivariate analysis; for example, the nature and severity of surgical complications in individual patients. Nevertheless, with these limitations in mind, our data do suggest that surgical ICU patients with severe sepsis may not benefit from adjuvant selenium supplementation with the dosage used in our study.

Dr Schomburg also suggests that selenium supplementation may be beneficial in patients with selenium deficiency [2], and refers to the earlier study of Angstwurm and colleagues [5] to support this assumption. Although this study reported selenium levels in patients included in the analysis, it did not provide clear evidence to confirm the assumption because selenium levels were similar at baseline between the study groups. The discrepancy between our results [1] and those of Angstwurm and colleagues [5] may be attributed to the differences in case mix. A recently completed multicenter study, the Placebo Controlled Trial of Sodium Selenite and Procalcitonin Guided Antimicrobial Therapy in Severe Sepsis (SISPCT, ClinicalTrials.gov NCT00832039), will hopefully provide further insight on this debate.

## Competing interests

The authors declare that they have no competing interests.
